# A Bayesian latent class extension of naive Bayesian classifier and its application to the classification of gastric cancer patients

**DOI:** 10.1186/s12874-023-02013-4

**Published:** 2023-08-21

**Authors:** Kimiya Gohari, Anoshirvan Kazemnejad, Marjan Mohammadi, Farzad Eskandari, Samaneh Saberi, Maryam Esmaieli, Ali Sheidaei

**Affiliations:** 1 Department of Biostatistics, Faculty of Medical Sciences, Tarbiat Modares University, Tehran, Iran; 2 HPGC Research Group, Department of Medical Biotechnology, Biotechnology Research Center, Pasteur Institute of Iran, Tehran, Iran; 3 Department of Statistics, Allameh Tabataba’i University, Tehran, Iran; 4 Department of Epidemiology and Biostatistics, School of Public Health, Tehran University of Medical Sciences, Tehran, Iran

**Keywords:** Naïve Bayesian classifier, Bayesian latent class analysis, Gibbs sampling, Expectation maximization algorithm, Gastric cancer

## Abstract

**Background:**

The Naive Bayes (NB) classifier is a powerful supervised algorithm widely used in Machine Learning (ML). However, its effectiveness relies on a strict assumption of conditional independence, which is often violated in real-world scenarios. To address this limitation, various studies have explored extensions of NB that tackle the issue of non-conditional independence in the data. These approaches can be broadly categorized into two main categories: feature selection and structure expansion.

In this particular study, we propose a novel approach to enhancing NB by introducing a latent variable as the parent of the attributes. We define this latent variable using a flexible technique called Bayesian Latent Class Analysis (BLCA). As a result, our final model combines the strengths of NB and BLCA, giving rise to what we refer to as NB-BLCA. By incorporating the latent variable, we aim to capture complex dependencies among the attributes and improve the overall performance of the classifier.

**Methods:**

Both Expectation-Maximization (EM) algorithm and the Gibbs sampling approach were offered for parameter learning. A simulation study was conducted to evaluate the classification of the model in comparison with the ordinary NB model. In addition, real-world data related to 976 Gastric Cancer (GC) and 1189 Non-ulcer dyspepsia (NUD) patients was used to show the model's performance in an actual application. The validity of models was evaluated using the 10-fold cross-validation.

**Results:**

The presented model was superior to ordinary NB in all the simulation scenarios according to higher classification sensitivity and specificity in test data. The NB-BLCA model using Gibbs sampling accuracy was 87.77 (95% CI: 84.87-90.29). This index was estimated at 77.22 (95% CI: 73.64-80.53) and 74.71 (95% CI: 71.02-78.15) for the NB-BLCA model using the EM algorithm and ordinary NB classifier, respectively.

**Conclusions:**

When considering the modification of the NB classifier, incorporating a latent component into the model offers numerous advantages, particularly within medical and health-related contexts. By doing so, the researchers can bypass the extensive search algorithm and structure learning required in the local learning and structure extension approach. The inclusion of latent class variables allows for the integration of all attributes during model construction. Consequently, the NB-BLCA model serves as a suitable alternative to conventional NB classifiers when the assumption of independence is violated, especially in domains pertaining to health and medicine.

**Supplementary Information:**

The online version contains supplementary material available at 10.1186/s12874-023-02013-4.

## Background

The Naive Bayes (NB) classifier is a well-established supervised algorithm in the field of Machine Learning (ML). Its simplicity and effectiveness in classification tasks have made it widely adopted across various domains [1, 2]. However, the NB classifier is built upon a fundamental assumption of conditional independence, wherein all feature pairs are considered mutually independent given the class variable [3]. In practical real-world scenarios, this assumption is frequently violated, resulting in a reduction in the algorithm's performance [4].

In the context of health and medical domains, the features employed in analysis often originate from diverse aspects related to the subjects under study [5]. These features can encompass symptoms in diagnostic scenarios or risk factors in the context of risk assessment. Consequently, the dependence among these features, even within a specific class, becomes inevitable. This dependency violates the assumption of conditional independence and calls for alternative approaches to effectively model and classify the data.

The issue of non-conditional independence in data has been addressed by various studies, proposing extensions of the Naive Bayes (NB) classifier [6]. These approaches can be classified into two major categories. Firstly, some studies focused on altering the features through subset selection or assigning weights to them [7–11]. These approaches involve a search strategy to identify the most relevant features that optimize the classification performance of NB. Feature selection methods aim to identify critical variables based on their contribution to classification and eliminate less influential ones [12]. Alternatively, feature weighting algorithms retain all variables in the model while assigning them importance weights [13–15]. However, these algorithms heavily rely on the characteristics of the observed data, and their results can vary accordingly. Moreover, the application of these methods is computationally demanding, as they pose NP-hard (NP-hard: Denoting a computational problem that is at least as difficult to solve as the hardest problems in the class of problems known as NP, which includes a wide range of challenging computational tasks) problems requiring extensive computational resources [13].

In an alternative approach, some studies have proposed expanding the structure of the Naive Bayes (NB) classifier to accommodate conditional independence. Examples of such methods include the Augmented Naive Bayes (ANB) [16, 17], Tree Augmented Naive Bayes (TAN) [18], extended Tree Augmented Naive Bayes (eTAN) [19], k-dependence Bayesian classifier [20], and Averaged One-Dependence Estimators (AODE) [21]. These algorithms share a common feature of augmenting the relationship set by introducing additional arcs between features. However, as more relationships are added to the original NB structure, the computational complexity increases. Hence, the challenge lies in striking a balance between the trade-off of increased relationships and computational complexity. Consequently, the search algorithms employed in this context face the same issue of being NP-hard [22].

An appealing alternative approach in extending the structure involves incorporating a latent variable into the model. By introducing a latent variable, we can effectively capture the correlation between features and enforce conditional independence within the structure [23–25]. The utilization of latent variables holds particular relevance in health and medical applications, especially in cases where the underlying causal mechanisms of diseases remain unknown. Additionally, latent variables find application in situations where the direct cause of a disease is not directly measurable, but certain observable variables can provide valuable insights into it [5]. Real medical data often involves complex interactions and relationships among various factors that influence health outcomes. The inclusion of latent variables provides a mechanism to capture these hidden factors, which may not be directly observable or measured [26, 27]. By incorporating latent variables into our models, we can account for unobserved factors that impact the observed features, leading to a more comprehensive understanding of the underlying mechanisms and improved predictive accuracy.

Defining a latent variable in the context of Naive Bayes (NB) requires careful consideration. Firstly, the placement of the latent variable within the structure determines its relationship with the features and class. For example, Langseth and Nielsen (2006) proposed a hierarchical NB model where class variables serve as the root, attributes act as leaf nodes, and multiple latent variables act as parents to the leaf nodes [28]. Calders and Verwer (2010) presented an NB model for discrimination-free classification, incorporating a single latent variable as the parent of the class variable [29]. Similarly, Alizadeh et al. (2021) introduced a multi-independent latent component extension of NB, featuring a latent variable as the parent of attributes and also linked to the class variable [23].

Additionally, defining the latent variable(s) requires careful consideration. The latent variable should encapsulate all relevant information from the attributes while assisting the NB structure in maintaining the assumption of conditional independence. Striking a balance between capturing the dependencies in the data and preserving the conditional independence assumption is essential in defining the latent variable(s).

This study introduces a novel approach by incorporating a latent variable as the parent of attributes, similar to the model proposed by Calders and Verwer. However, our proposed model offers reduced complexity compared to the previous approach. The latent variable is defined using Bayesian Latent Class Analysis (BLCA), providing flexibility in modeling. As a result, our final model combines elements of both Naive Bayes (NB) and BLCA, and we refer to it as NB-BLCA. To learn the model's parameters, we provide two options: the Expectation-Maximization (EM) algorithm and the Gibbs sampling approach. A comprehensive simulation study is conducted to assess the classification performance of the proposed model. Furthermore, we apply the model to real-world data, specifically in classifying patients as either GC or NUD based on their attributes. By employing the NB-BLCA model, we aim to enhance classification accuracy while effectively capturing latent dependencies within the data, contributing to improved decision-making in healthcare settings.

## Material and methods

### Naïve Bayesian classifier

Suppose in a classification problem, the levels of target variable $C$ indicate the different classes. For instance, $C$ could be the disease status indicator. In this example, the $C$ levels indicate the disease's presence or absence. Another example could be a physician's diagnosed stages of GC patients. In such examples, we are interested in exploring the prediction power of a set of attributes $(X_{1},\dots ,{X}_{m})$ for accurately detecting $C$ levels. In an NB classifier framework, we assume the attributes $(X_{1},\dots ,{X}_{m})$ are conditionally independent given the information about class variable $C$. Therefore, we aim to find the level $c$ of the class variable $C$ which maximizes the posterior probability of this variable given the observed values of attributes:

1 $$\underset{c\in C}{\arg\;\mathit{max}\;}P(C\vert x_1,\dots,x_m)$$

Using the Bayes rule for this posterior probability, we have:

2 $$P\left(C|{x}_{1},\dots ,{x}_{m}\right)=\frac{P(C)P({x}_{1},\dots ,{x}_{m}|C)}{P({x}_{1},\dots ,{x}_{m})}$$

As we mentioned before, the primary assumption of NB is conditional independency between attributes given the class variable. Therefore equation (2) could be rewritten as:

3 $$P\left(C|{x}_{1},\dots ,{x}_{m}\right)=\frac{\prod_{i=1}^{m}P\left({x}_{i}|C\right)P(C)}{{\sum }_{c}\prod_{i=1}^{m}P\left({x}_{i}|C=c\right)P(C=c)}$$

In equation (3), the denominator is constant for all the possible values of class variable $C$. Hence we could eliminate it and find the best class according to the below formula:

4 $$\underset{c\in C}{\arg\;max\;}P(C)\prod_{i=1}^mP(x_i\vert C)$$

Therefore we allocate the subjects to the class variable levels, which are maximized according to their attributes.

### Bayesian latent class analysis

BLCA is a model-based clustering that finds explicitly unobserved homogenous subgroups among the total population and uses the Bayesian paradigm in this manner [30, 31]. This study introduces a version of Bayesian Latent Class Analysis (BLCA) specifically tailored for binary attributes while accommodating a multinomial distributed class variable. While it is possible to generalize the method for multinomial attributes or predictors, it requires the use of binary indicator variables, which is a common practice in various statistical applications such as regression. By employing this approach, for a dependent factor variable with q levels, one can include q-1 binary indicators, with each indicator representing a specific level of the original dependent variable by taking the value 1 and 0 for the other levels. The elimination of the last level is necessary to avoid redundancy. However, it is important to note that the binary version of BLCA often suffices for many health and medical applications.

Suppose we express the attributes by an M-dimensional vector-valued ${\varvec{X}}=({{\varvec{X}}}_{1},\dots ,{{\varvec{X}}}_{N})$, where these come from G sub-populations. The sub-populations are typically referred to as classes or components. Therefore, we have two sets of parameters. A G-dimensional vector ${\varvec{\tau}}=({\tau }_{1},\dots ,{\tau }_{G})$, including parameters for prior belief in the proportions of each class. In addition, a matrix ${\varvec{\theta}}$ with dimension $G\times M$ for item probability of all classes. In this way, all elements ${\varvec{\tau}}$ are equal or greater than 0 and $\sum_{g=1}^{G}{\tau }_{g}=1$ and ${\theta }_{gm}$ is the probability of ${X}_{im}=1$ given the information about membership of group $g$ for any $i\in 1,\dots ,N$ of individuals in the study. Hence, we have $P\left({X}_{im}|{\theta }_{gm}\right)={\theta }_{gm}^{{X}_{im}}{(1-{\theta }_{gm})}^{1-{X}_{im}}$ for ${X}_{im}\in [\mathrm{0,1}]$, according to the definition of Bernoulli distribution.

If we make a naïve Bayes assumption of conditional independence of observations given the group membership, we can express the $P\left({{\varvec{X}}}_{i}|{{\varvec{\theta}}}_{g}\right)={\prod }_{m=1}^{M}P({X}_{im}|{\theta }_{gm})$ and the distribution of all ${{\varvec{X}}}_{i}$ s are:

5 $$P\left({{\varvec{X}}}_{i}|{\varvec{\theta}},{\varvec{\tau}}\right)=\sum_{g=1}^{G}{\tau }_{g}P({{\varvec{X}}}_{i}|{{\varvec{\theta}}}_{g})$$

The actual values for parameters ${\varvec{\theta}}$ and ${\varvec{\tau}}$ are unknown, and we suppose prior information about them. Therefore, the direct calculation of equation 5 is not feasible. In application, we introduce a set ${\varvec{Z}}=\left({{\varvec{Z}}}_{1},\dots ,{{\varvec{Z}}}_{N}\right)$ where each ${{\varvec{Z}}}_{i}=({Z}_{i1},\dots ,{Z}_{iG})$ is a vector representing the actual class membership of ${{\varvec{X}}}_{i}$. In this manner, ${Z}_{ig}=1$ if individual $i$ belongs to subgroup $g$ and 0 for otherwise. The new task is to find the best values for ${\varvec{Z}},$ which maximize the posterior probability of class membership, including the ${\varvec{Z}}$ parameters.

The complete density of observed variables ${{\varvec{X}}}_{i}$ and missing values ${{\varvec{Z}}}_{i}$ is:

$$P\left({{\varvec{X}}}_{i},{{\varvec{Z}}}_{i}|{\varvec{\tau}},{\varvec{\theta}}\right)=\prod_{g=1}^{G}{[{\tau }_{g}P({{\varvec{X}}}_{i}|{{\varvec{\theta}}}_{g})]}^{{Z}_{ig}}$$

Using the Bayes theorem leads to the posterior probability of ${{\varvec{Z}}}_{i}$, class membership for observation $i$, as:

$$P\left({{\varvec{Z}}}_{i}|{{\varvec{X}}}_{i},{\varvec{\tau}},{\varvec{\theta}}\right)=\prod_{g=1}^{G}{[\frac{{\tau }_{g}P({{\varvec{X}}}_{i}|{{\varvec{\theta}}}_{g})}{\sum_{h=1}^{G}{\tau }_{h}P({{\varvec{X}}}_{i}|{{\varvec{\theta}}}_{h})}]}^{{Z}_{ig}}$$

The drawback of unknown actual values for parameters ${\varvec{\theta}}$ and ${\varvec{\tau}}$ still exist. An iterative approach that updates the prior information of these parameters in each step according to the observed data is proposed to achieve the best posterior distribution. In this regard, we assume conjugate prior distribution $Beta({\alpha }_{gm},{\beta }_{gm})$ for binary variables ${\varvec{\theta}}$, and $Dirichlet({\varvec{\delta}})$ for multinomial variables ${\varvec{\tau}}$. Note that hyperparameters ${\alpha }_{gm}$ and ${\beta }_{gm}$ for Beta prior distributions, specify the item response probabilities of attributes $m$ in class $g$. In the same manner, hyperparameter ${\varvec{\delta}}=({\delta }_{1},\dots ,{\delta }_{G})$ specify the share of each class from the total samples.

Supposing these prior distributions for ${\varvec{\theta}}$ and ${\varvec{\tau}}$ we have:

$$P({\varvec{\tau}}|{\varvec{\delta}})\propto \prod_{g=1}^{G}{\tau }_{g}^{{\delta }_{g}-1}$$

$$P({\theta }_{gm}|{\alpha }_{gm},{\beta }_{gm})\propto {\theta }_{gm}^{{\alpha }_{gm}-1}{(1-{\theta }_{gm})}^{{\beta }_{gm}-1}$$

For each $g\in [1,\dots ,G]$ and $m\in [1,\dots ,M]$. These assumptions lead to the joint posterior distribution ${\varvec{\tau}}$ and ${\varvec{\theta}}$ as:

$$P\left({\varvec{\tau}},{\varvec{\theta}}\right)\propto \prod_{i=1}^{N}P\left({{\varvec{X}}}_{i},{{\varvec{Z}}}_{i}|{\varvec{\tau}},{\varvec{\theta}}\right)P\left({\varvec{\theta}}\right)P\left({\varvec{\tau}}\right)=\prod_{i=1}^{N}\prod_{g=1}^{G}{\tau }_{g}^{{Z}_{ig}+{\delta }_{g}-1}\prod_{m=1}^{M}{\theta }_{gm}^{{X}_{im}{Z}_{ig}+{\alpha }_{gm}-1}{(1-{\theta }_{gm})}^{\left(1-{X}_{im}\right){Z}_{ig}+{\beta }_{gm}-1}$$

In the following parts, we present two well-known iterative approaches for parameter estimation. These are the EM algorithm and Gibbs sampling method.

### The EM algorithm for BLCA

This algorithm follows an iterative process that continues until convergence is achieved, iteratively refining the results. The algorithm consists of two steps that are repeated in each iteration. In the first step, the algorithm calculates the expectation of the logarithm posterior probability. This step involves estimating the probabilities associated with each parameter based on the available data. In the second step, the algorithm determines the parameter values that maximize the expectation function obtained in the previous step. This maximization step involves adjusting the parameter values to optimize the fit of the model to the data [32]. To initiate the algorithm, an initial guess of the parameter values is required for the first iteration. However, regardless of the initial values chosen, the algorithm is guaranteed to converge to the actual values of the parameters. The number of iterations required for convergence may vary depending on the specific dataset and initial values chosen.

By iteratively performing these two steps, the algorithm refines the parameter estimates, improving the accuracy and performance of the model until a satisfactory level of convergence is achieved [33]. If we show the values of the parameters ${\varvec{\tau}}$ and ${\varvec{\theta}}$ in steps $k$ by ${{\varvec{\tau}}}^{(t)}$ and ${{\varvec{\theta}}}^{(t)}$, respectively the expected function in E-step for a BLCA is:

$$\boldsymbol{Q}(\boldsymbol{\theta},\boldsymbol{\tau}|\boldsymbol{\theta}^{(t)},\boldsymbol{\tau}^{(t)}):=\boldsymbol{E}[\log\boldsymbol{P}(\boldsymbol{\theta},\boldsymbol{\tau}|\boldsymbol{X},\boldsymbol{Z})|\boldsymbol{X},{\theta}^{(t)},\boldsymbol{\tau}^{(t)}]$$

In the M-step, we update the parameters as follows:

$${{\varvec{\theta}}}^{(t+1)}=\underset{{\varvec{\theta}}\in\Theta }{\mathrm{arg}maxQ\left({\varvec{\theta}},{\varvec{\tau}}|{{\varvec{\theta}}}^{(t)},{{\varvec{\tau}}}^{(t)}\right)}$$

$${{\varvec{\tau}}}^{(t+1)}=\underset{{\varvec{\tau}}\in \mathrm{T}}{\mathrm{arg}maxQ\left({\varvec{\theta}},{\varvec{\tau}}|{{\varvec{\theta}}}^{(t)},{{\varvec{\tau}}}^{(t)}\right)}$$

Here the $\Theta$ and $\mathrm{T}$ are parameter space for ${\varvec{\theta}}$ and ${\varvec{\tau}},$ respectively. For all item response probability and class proportions, we have $\Theta ={[\mathrm{0,1}]}^{G\times M}$ and $\mathrm{T}={[\mathrm{0,1}]}^{G}$ given $\sum_{g=1}^{G}{\tau }_{g}=1$.

It has been shown that the practical formulations for these steps are [34]:

E-step:

$${Z}_{ig}^{(t+1)}=\frac{{\tau }_{g}^{\left(t\right)}P({{\varvec{X}}}_{i}|{{\varvec{\theta}}}_{g}^{\left(t\right)})}{\sum_{h=1}^{G}{\tau }_{h}^{\left(t\right)}P({{\varvec{X}}}_{i}|{{\varvec{\theta}}}_{h}^{(t)})}$$

M-step:

$${\theta }_{gm}^{(t+1)}=\frac{\sum_{i=1}^{N}{X}_{im}{Z}_{ig}^{(t+1)}+{\alpha }_{gm}-1}{\sum_{i=1}^{N}{Z}_{ig}^{(t+1)}+{\alpha }_{gm}+{\beta }_{gm}-2}$$

$${\tau }_{g}^{(t+1)}=\frac{\sum_{i=1}^{N}{Z}_{ig}^{(t+1)}+{\delta }_{g}-1}{N+\sum_{h=1}^{G}{\delta }_{h}-G}$$

### The Gibbs sampling for BLCA

As we already mentioned, calculating the joint posterior distribution of parameters ${\varvec{\tau}}$ and ${\varvec{\theta}}$ and unobserved class membership ${\varvec{Z}}$ is directly impossible. However, determining the class membership of samples is possible in the case of knowing the parameter values. Gibbs sampling is a Markov Chain Monte Carlo (MCMC) method that simplifies such issues and, instead of using the joint distribution, iteratively draws samples from the conditional distributions using the Markov property. These samples reflect the properties of the accurate joint posterior distribution [35].

The following steps are the practical approach for handling a BLCA using the Gibbs sampling:

1. Set initial values for parameters ${\varvec{\tau}}$ and ${\varvec{\theta}}$ and randomly assign each observation to a class. Although this step plays a crucial role in determining the convergence speed of the algorithm, it is important to provide guidance on how users can specify the initial values effectively. In our proposed method, one approach for specifying initial values is to use random initialization, which allows for exploration of different parts of the parameter space. This can help avoid potential biases that may arise from using fixed initial values. Additionally, users may consider conducting sensitivity analyses by running the algorithm multiple times with different initializations to assess the stability of the results.
2. Considering the conjugate prior of Beta distribution, generate elements of ${{\varvec{\theta}}}^{(t)}$ randomly from the following distribution:
  $$\theta_{gm}^{(t)}\sim Beta(\sum_{i=1}^NX_{im}Z_{ig}^{(t-1)}+\alpha_{gm},\sum_{i=1}^NZ_{ig}^{\left(t-1\right)}\left(1-X_{im}\right)+\beta_{gm})$$
3. Considering the conjugate prior of Dirichlet distribution, generate elements of ${{\varvec{\tau}}}^{(k+1)}$ randomly from the following distribution:
  $${{\varvec{\tau}}}^{(t)}\sim Dirichlet(\sum_{i=1}^{N}{Z}_{i1}^{\left(t-1\right)}+{\delta }_{1},\dots ,\sum_{i=1}^{N}{Z}_{iG}^{(t-1)}+{\delta }_{G})$$
4. Consider the generated values of parameters and assign the individuals to classes randomly from a multinomial distribution according to their observed attributes ${{\varvec{X}}}_{i}$ which specify the posterior probabilities of membership in the classes:
  $${{\varvec{Z}}}_{i}^{(t)}\sim Multinomial(1,\frac{{\tau }_{1}^{\left(t\right)}P\left({{\varvec{X}}}_{i}|{{\varvec{\theta}}}_{1}^{\left(t\right)}\right)}{\sum_{h=1}^{G}{\tau }_{h}^{\left(t\right)}P\left({{\varvec{X}}}_{i}|{{\varvec{\theta}}}_{h}^{\left(t\right)}\right)},\dots ,\frac{{\tau }_{G}^{\left(t\right)}P({{\varvec{X}}}_{i}|{{\varvec{\theta}}}_{G}^{(t)})}{\sum_{h=1}^{G}{\tau }_{h}^{\left(t\right)}P({{\varvec{X}}}_{i}|{{\varvec{\theta}}}_{h}^{(t)})})$$
5. Repeat steps 2 to 4 until making sure about convergence.

After running the Gibbs sampling, like all other MCMC methods, it is essential to check if the chain converged using the statistical criteria and trace plots. In addition, burn-in and thinning are necessary [36].

### NB-BLCA

**Fig. 1:**
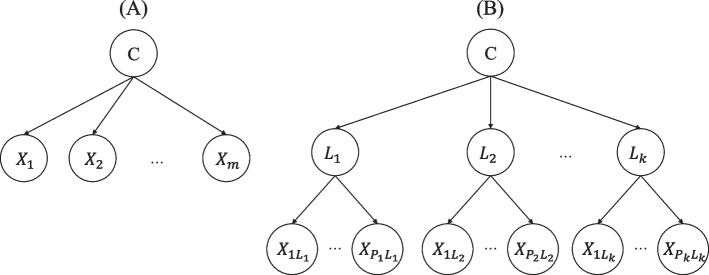
The Naïve Bayesian classifier (**A**) and proposed model network (**B**) structures

In this study, we present an extension of the NB classifier that uses BLCA to impose conditional independence assumptions on the structure of the model. NB and BLCA assume the Naïve assumption of conditional independence assumption given the information of class variable. In contrast to NB, which only requires this assumption for efficient classification, The BLCA model estimates the parameter values considering this purpose. The presentation of the NB classifier and our proposed model are depicted in Fig. 1, parts A and B, respectively. In this figure, the latent class of BLCA is shown by ${L}_{i} [i=1,\dots ,K]$ to differentiate from classes of the primary outcome $C$. Remember that latent class $L$ is unobserved, but the class variable $C$ is observable.

In the NB-BLCA model, the only child node of class variable $C$ is the latent class variables ${L}_{i}$. Therefore the posterior density in equation 3 could be reformed to:

$$P\left(C|L\right)=\frac{{\prod }_{i=1}^{k}P\left({L}_{i}|C\right)P(C)}{{\sum }_{c}\prod_{i=1}^{k}P({L}_{i}|C=c)P(C=c)}$$

As the latent class variables ${L}_{i}$ come from a mixture distribution with parameters $({\varvec{\tau}},{\varvec{\theta}},{\varvec{Z}}$), the calculation of this posterior probability is not straightforward. However, the generalized forms of the EM algorithm and Gibbs sampling in the previous sections enable us to predict class membership $C$ due to information about the latent class assignment ${L}_{i}$ concluded from the observed attributes.

### Adjusting EM algorithm for NB-BLCA

In order to explain the EM algorithm for an NB-BLCA, we should define the following parameters:

The parameter $q\left(c\right)$ is the probability of seeing the level $c$ of the class variable. Hence, it is subject to constraints $q(c)\ge 0$ and $\sum q(c)=1$ for all the possible levels of this variable.

The parameter ${q}_{i}(l|c)$ for any $i=1,\dots ,K$ is the probability of latent class $i$ taking value $l$, conditioned on the class $c$. This parameter is subject to constraints ${q}_{i}(l|c)\ge 0$ and $\sum {q}_{i}\left(l|c\right)=1$ for all levels of class and latent class variables.

**Fig. 2:**
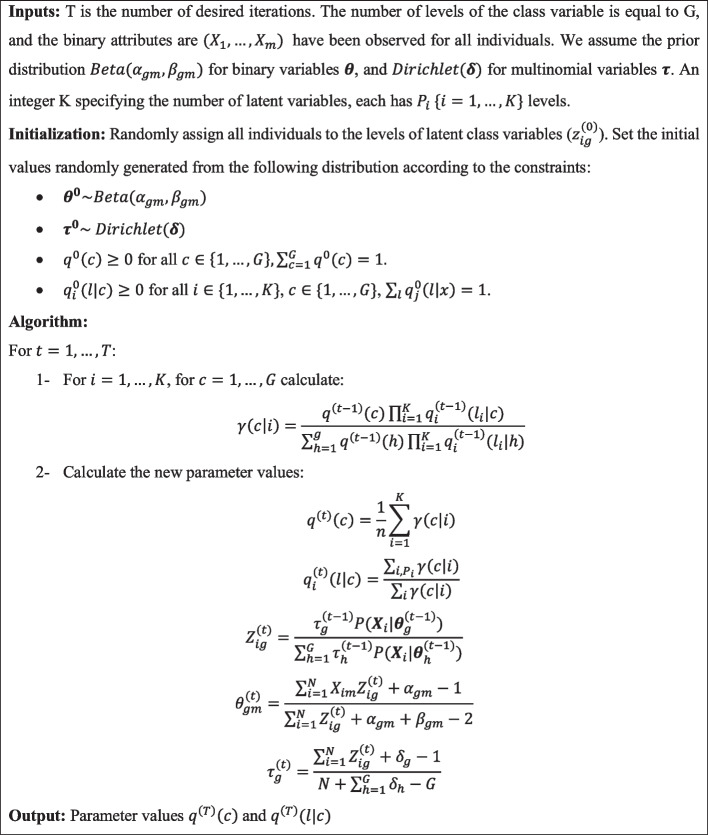
The EM Algorithm for the NB-BLCA model

The practical formulations of the EM algorithm are presented in Fig. 2. The algorithm estimates latent class variables membership using the attributes and then estimate the posterior probability of class membership of the target variable.

### Adjusting Gibbs sampling for NB-BLCA

**Fig. 3:**
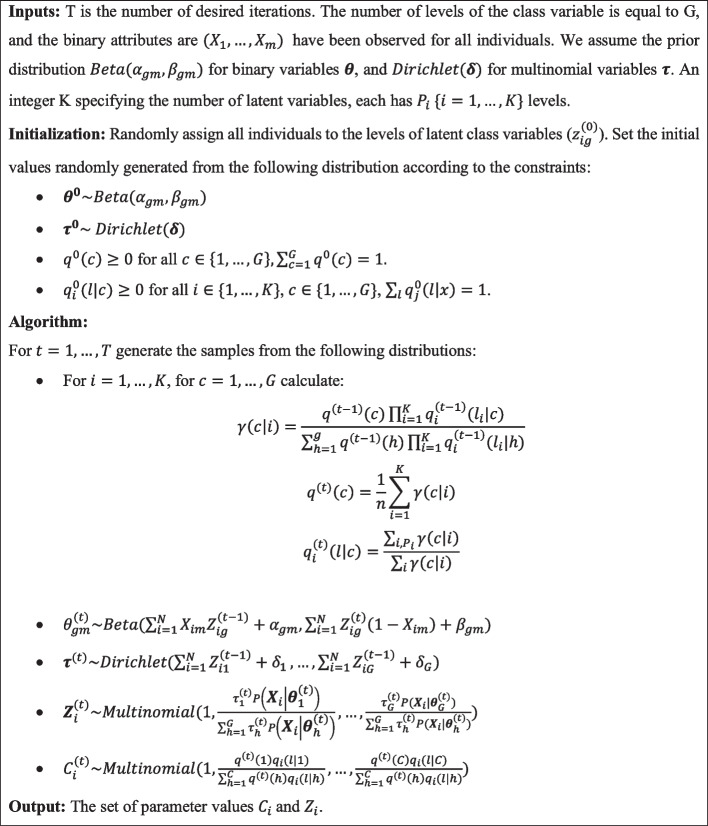
The Gibbs sampler Algorithm for the NB-BLCA model

The Gibbs sampler simplifies a complex joint posterior distribution into a set of steps, including generating samples from the conditional distributions. We explained how to generate latent class membership samples for a BLCA problem in 5 steps. The added task of generating samples for the NB part of NB-BLCA is quickly done by adding an extra step. The sample generation could be done from a multinomial (if the class variable has more than two categories) or binomial distribution (the class variable only includes two levels). The practical formulations of the Gibbs sampler are presented in Fig. 3.

### Simulation study

We conducted a simulation study to evaluate the predictive performance of our model compared to a simple NB model. Furthermore, we included two alternative approaches that have been suggested to improve the correct classification of NB when the conditional assumption is violated. These approaches are Averaged one-dependence estimators (AODE), proposed by Webb et al. [21], and Hill-climbing tree augmented naive Bayes (TAN-HC), proposed by Keogh and Pazzani [37].

To generate the datasets, we utilized the Iterative Proportional Fitting Procedure (IPFP), originally proposed by Deming and Stephan in 1940 as an algorithm aimed at minimizing the Pearson chi-squared statistic [38]. The details of this method, as described by Suesse et al. [39], can be found in the 'mipfp' R package developed by Barthélemy and Suesse [40]. Using this method we were able to simulate multivariate Bernoulli distributions assuming the Hypothetical Marginal Probabilities (HMP) of each variable and a matrix that includes the Odds Ratio (OR) of all pairs of variables.

The elements of the HMP vector were randomly generated from a uniform distribution between 0 and 1 (${\mathrm{HMP }}_{i}\sim U(\mathrm{0,1})$) for each iteration. Similarly, the elements of the paired OR matrix were randomly generated from a uniform distribution within the range of 0.25 and 4 (${OR}_{ij}\sim U\left(\mathrm{0.25,4}\right) for i\ne j$). To reduce computational complexity, we generated the feature variables in batches of 5 dimensions. Consequently, for scenarios involving only 5 features, we generated a single batch. For scenarios with 10 features, we generated 2 batches, and so on.

The response class variable $Z$ was generated using a logistic regression approach. We assumed a regression coefficient of 2 ($\beta =2$) for all feature variables and applied the inverse logit transformation to their linear combination to calculate the probability of belonging to class 1. Additionally, a random error term from a Gaussian distribution with mean parameter 0 and standard deviation parameter 4 was added to this linear combination. The intercept coefficient ($\alpha$) of the logistic regression served as a tuning parameter for specifying the marginal probability of the class variable.

$$Z=\alpha +\beta \sum_{i=1}^{p}{X}_{i}+N(0,\sigma =4)$$

$$P= \frac{1}{1+{e}^{Z}}$$

$$Y\sim Binomial(P)$$

Finally, the values of the response variable were generated from a Binomial distribution, taking into account the calculated probabilities.

We assumed marginal probabilities of 0.3, 0.5, and 0.7 for the class variable to explore their effect on the model's performance. To assess the impact of sample size on the model's performance, we considered samples consisting of 500, 1000, and 2000 subjects. Furthermore, we generated scenarios with 5, 10, and 20 feature variables.

For all algorithms, we used 70% of the randomly selected data as a training dataset, while the remaining 30% was used to evaluate algorithm performance. The validity of the algorithms was measured by calculating the mean values of sensitivity (recall), specificity, positive predictive value (precision), negative predictive value, and precision across 1000 replicates."

### Real-world data application

In this section, we used multicenter hospital-based data to demonstrate the application of the model in a real-world example. This data was related to 976 GC and 1189 NUD patients referred to the national cancer institute of Iran (NCII) from July 2003 to Jan 2020. Trained technicians interviewed each participant at the time of recruitment using a structured questionnaire after accepting enrolment in the study. The questionnaire includes 64 attributes in the five subdomains, demographic variables, dietary habits, self-reported medical status, narcotics use, and SES indicators. All the predictors were recoded into binary variables, and the list, including their names and levels, is available in Supplementary Table 1.

We fitted the NB classifier, NB-BLCA using the EM algorithm, and NB-BLCA using Gibbs sampler to data. A random sample with a proportion of 70% sample size was selected to train the models. The model's validity and prediction ability were explored using the other 30% of subjects. The identical measurements in the simulation section were calculated and reported.

## Results

In the simulation study, we compared the sensitivity, specificity, positive predictive value, negative predictive value, and precision of the ordinary Naive Bayes (NB) classifier, NB-BLCA, and other alternative models. Tables 1, 2 and 3 present these performance metrics for different scenarios, considering varying marginal probabilities of the class variables (0.3, 0.5, and 0.7) and different numbers of predictors.

**Table 1:** Comparison of Naive Bayes classifier and alternative approach: marginal probability of class variable = 0.3

Model	Number of predictors	Sample size	Sensitivity	Specificity	Positive predictive value	Negative predictive value	Precision
NB	5	500	39.21	86.81	58.13	76.05	58.13
AODE	5	500	36.78	88.82	60.64	75.71	60.64
TAN	5	500	38.17	87.81	59.31	75.93	59.31
NB-BLCA (EM)	5	500	35.46	90.69	64.13	75.69	64.13
NB	5	1000	38.98	86.7	57.76	75.96	57.76
AODE	5	1000	35.8	88.76	59.8	75.43	59.8
TAN	5	1000	37.61	87.61	58.56	75.73	58.56
NB-BLCA (EM)	5	1000	33.25	90.74	62.45	75.06	62.45
NB	5	2000	38.75	86.64	57.56	75.86	57.56
AODE	5	2000	35.71	88.51	59.23	75.32	59.23
TAN	5	2000	37.36	87.49	58.24	75.61	58.24
NB-BLCA (EM)	5	2000	31.74	90.81	61.35	74.6	61.35
NB	10	500	49.48	87.13	63.48	79.37	63.48
AODE	10	500	49.86	88.27	65.81	79.7	65.81
TAN	10	500	49.92	87.69	64.73	79.61	64.73
NB-BLCA (EM)	10	500	66.38	92.52	80.08	86.04	80.08
NB	10	1000	48.47	87.26	63.19	79.06	63.19
AODE	10	1000	48.34	88.1	64.71	79.18	64.71
TAN	10	1000	48.51	87.51	63.66	79.12	63.66
NB-BLCA (EM)	10	1000	59.43	91.62	76.19	83.46	76.19
NB	10	2000	48.49	87.1	62.94	79	62.94
AODE	10	2000	48.12	87.74	63.97	79.01	63.97
TAN	10	2000	48.41	87.2	63.08	79	63.08
NB-BLCA (EM)	10	2000	54.27	90.92	72.96	81.58	72.96
NB	20	500	59.13	89.33	71.18	83.12	71.18
AODE	20	500	62.15	90.31	74.12	84.32	74.12
TAN	20	500	60.33	89.8	72.52	83.61	72.52
NB-BLCA (EM)	20	500	98.73	99.69	99.31	99.43	99.31
NB	20	1000	57.66	89.26	70.54	82.57	70.54
AODE	20	1000	59.26	89.82	72.21	83.2	72.21
TAN	20	1000	57.91	89.4	70.91	82.67	70.91
NB-BLCA (EM)	20	1000	97.63	99.33	98.48	98.95	98.48
NB	20	2000	57.29	89.12	70.04	82.47	70.04
AODE	20	2000	58.14	89.44	70.96	82.81	70.96
TAN	20	2000	57.31	89.13	70.07	82.48	70.07
NB-BLCA (EM)	20	2000	95.86	98.8	97.24	98.18	97.24

**Table 2:** Comparison of Naive Bayes classifier and alternative approach: marginal probability of class variable = 0.5

Model	Number of predictors	Sample size	Sensitivity	Specificity	Positive predictive value	Negative predictive value	Precision
NB	5	500	64.5	67.25	66.69	65.72	66.69
AODE	5	500	65.28	67.51	67.16	66.33	67.16
TAN	5	500	64.96	67.43	66.97	66.08	66.97
NB-BLCA (EM)	5	500	65.75	68.97	68.32	67.07	68.32
NB	5	1000	64.36	67.06	66.39	65.71	66.39
AODE	5	1000	64.73	67.24	66.69	66.02	66.69
TAN	5	1000	64.63	67.07	66.51	65.87	66.51
NB-BLCA (EM)	5	1000	64.93	68.12	67.25	66.33	67.25
NB	5	2000	64.39	66.82	66.34	65.59	66.34
AODE	5	2000	64.43	67.1	66.57	65.73	66.57
TAN	5	2000	64.34	66.96	66.42	65.61	66.42
NB-BLCA (EM)	5	2000	64.39	67.69	66.77	65.75	66.77
NB	10	500	70.26	71.57	71.31	70.67	71.31
AODE	10	500	71.08	72.65	72.33	71.57	72.33
TAN	10	500	70.81	72.26	71.97	71.24	71.97
NB-BLCA (EM)	10	500	79.43	82.61	82.16	80.19	82.16
NB	10	1000	70.08	71.25	71	70.45	71
AODE	10	1000	70.58	71.84	71.57	70.98	71.57
TAN	10	1000	70.28	71.41	71.17	70.64	71.17
NB-BLCA (EM)	10	1000	76.36	79.27	78.75	77.12	78.75
NB	10	2000	69.95	70.95	70.67	70.34	70.67
AODE	10	2000	70.26	71.26	71	70.66	71
TAN	10	2000	69.98	71.01	70.73	70.38	70.73
NB-BLCA (EM)	10	2000	73.8	76.62	75.96	74.64	75.96
NB	20	500	75.97	76.79	76.62	76.22	76.62
AODE	20	500	77.43	78.88	78.6	77.82	78.6
TAN	20	500	76.77	77.59	77.43	77.02	77.43
NB-BLCA (EM)	20	500	99.01	99.36	99.36	99.01	99.36
NB	20	1000	75.43	76.19	76.03	75.63	76.03
AODE	20	1000	76.24	77.34	77.11	76.51	77.11
TAN	20	1000	75.7	76.42	76.27	75.89	76.27
NB-BLCA (EM)	20	1000	98.13	98.62	98.61	98.15	98.61
NB	20	2000	75.39	75.99	75.89	75.52	75.89
AODE	20	2000	75.83	76.64	76.49	76	76.49
TAN	20	2000	75.41	76.02	75.92	75.54	75.92
NB-BLCA (EM)	20	2000	96.92	97.58	97.56	96.94	97.56

When the marginal probability of the class variable is set to 0.3 and the number of predictors is low (5 attributes), the sensitivity of all models is relatively lower, failing to exceed 50%. However, as the sample size increases, the sensitivity improves. Even in the scenario with the highest sample size of 2000, the sensitivity remains below 50%. This indicates that all algorithms are sensitive to the lower rate of events in the data. It is worth noting that both increasing the number of predictors and the marginal probability of the class variables enhance the sensitivity of the models.

In all scenarios, except for the marginal probability of the class variable 0.7 when the number of predictors is 5, the precision of our proposed model (NB-BLCA) is higher compared to the other approaches. This indicates that our model performs better in terms of correctly identifying positive instances among the predicted ones.

When the marginal probability of the class variable is low (0.3) and the number of predictors is less than 20, the superiority of our model is based on higher specificity. Increasing the number of predictors also leads to a greater increase in the sensitivity of our model compared to the other approaches. This trend is observed consistently across the different scenarios (as shown in Tables 2 and 3).

**Table 3:** Comparison of Naive Bayes classifier and alternative approach: marginal probability of class variable = 0.7

Model	Number of predictors	Sample size	Sensitivity	Specificity	Positive predictive value	Negative predictive value	Precision
NB	5	500	85.99	38.59	75.81	56.38	75.81
AODE	5	500	88.17	35.32	75.27	58.62	75.27
TAN	5	500	87.08	37.15	75.6	57.46	75.6
NB-BLCA (EM)	5	500	89.84	33.82	75.17	60.56	75.17
NB	5	1000	85.99	38.65	75.8	56.26	75.8
AODE	5	1000	88.21	34.98	75.16	58.03	75.16
TAN	5	1000	87.06	36.96	75.51	57.09	75.51
NB-BLCA (EM)	5	1000	90.06	32.09	74.67	59.26	74.67
NB	5	2000	85.87	38.67	75.76	56.05	75.76
AODE	5	2000	88.22	34.61	75.04	57.96	75.04
TAN	5	2000	87.02	36.75	75.43	56.9	75.43
NB-BLCA (EM)	5	2000	90.41	30.56	74.27	58.84	74.27
NB	10	500	86.26	50.37	79.44	62.46	79.44
AODE	10	500	87.23	50.59	79.69	64.24	79.69
TAN	10	500	86.77	50.72	79.65	63.5	79.65
NB-BLCA (EM)	10	500	90.28	66.73	85.82	75.7	85.82
NB	10	1000	86.5	49.01	79.13	62.06	79.13
AODE	10	1000	87.23	48.7	79.16	63.2	79.16
TAN	10	1000	86.71	49.01	79.17	62.41	79.17
NB-BLCA (EM)	10	1000	89.47	59.65	83.24	71.79	83.24
NB	10	2000	86.39	48.7	78.99	61.76	78.99
AODE	10	2000	87.09	48.07	78.92	62.68	78.92
TAN	10	2000	86.56	48.51	78.96	61.96	78.96
NB-BLCA (EM)	10	2000	89.05	54.23	81.29	68.98	81.29
NB	20	500	88.79	59.25	83.13	70.11	83.13
AODE	20	500	89.44	62.7	84.44	72.5	84.44
TAN	20	500	89.25	60.34	83.58	71.37	83.58
NB-BLCA (EM)	20	500	99.4	98.97	99.54	98.67	99.54
NB	20	1000	88.62	57.86	82.58	69.35	82.58
AODE	20	1000	88.95	59.71	83.27	70.62	83.27
TAN	20	1000	88.74	58.05	82.66	69.64	82.66
NB-BLCA (EM)	20	1000	98.76	97.65	98.95	97.24	98.95
NB	20	2000	88.65	57.45	82.45	69.21	82.45
AODE	20	2000	88.82	58.48	82.83	69.91	82.83
TAN	20	2000	88.66	57.45	82.45	69.23	82.45
NB-BLCA (EM)	20	2000	97.94	95.63	98.06	95.38	98.06

Similar to many classification algorithms, the performance of NB, AODE, TAN, and our proposed model is influenced by the prevalence of the outcome, with a lower rate of events having a significant impact on the sensitivity of these models.

Overall, the results demonstrate that the performance of the models is affected by the marginal probability of the class variable, the number of predictors, and the prevalence of the outcome. Our proposed model (NB-BLCA) shows favorable precision and specificity, particularly in scenarios with low marginal probability and a smaller number of predictors.

These findings highlight the importance of considering these factors when applying classification algorithms and emphasize the potential benefits of our proposed model in handling such scenarios.

In Table 4, we present the results of comparing the models' predictions for real world data (classification of patients into GC or NUD groups). All models showed a significant improvement in prediction accuracy (*P*-value < 0.001). Among the models, the NB-BLCA model utilizing the Gibbs sampler achieved the highest accuracy of 87.77 (84.87-90.29), according to the 95% confidence interval. Notably, this confidence interval did not overlap with the intervals of the other two models, indicating a statistically significant increase in prediction accuracy.

**Table 4:** Comparison between predictive indices of NB-BLCA models and ordinary NB in real-world data of GC patients

	**Model**		
**Index**	**NB-BLCA** **(EM algorithm)**	**NB-BLCA** **(Gibbs sampling)**	**NB classifier**
Accuracy (95% CI)	77.22 (73.64-80.53)	87.77 (84.87-90.29)	74.71 (71.02-78.15)
No information rate (NIR)	63.32	50.92	53.43
P-Value [Accuracy > NIR]	<0.001	<0.001	<0.001
Kappa	0.53	0.76	0.49
Mcnemar's Test P-Value	<0.001	<001	0.74
Sensitivity	81.28	82.89	71.94
Specificity	74.87	92.83	77.12
Pos Pred Value	65.2	92.31	73.26
Neg Pred Value	87.35	83.95	75.93
Balanced Accuracy	78.07	87.86	74.53

Additionally, the Gibbs sampler-based NB-BLCA model demonstrated a higher Kappa value compared to the other approaches. This indicates that the model correctly classified patients with a 76% higher accuracy than random assignment. Furthermore, when performing McNemar's test for the NB classifier, the result was not significant (*p*-value = 0.74), suggesting that the NB approach did not yield a substantial improvement.

While the NB-BLCA model had a lower specificity (74.87) compared to NB (77.12), it exhibited a significantly higher sensitivity. The increased sensitivity indicates a better ability to correctly identify positive cases. Overall, the NB-BLCA model employing the Gibbs sampler outperformed the other two alternatives in terms of prediction accuracy and various performance metrics.

## Discussion

We presented a modified version of the ordinary NB classifier called NB-BLCA, which can enhance the model's prediction performance. In addition, we suggested two methods, Gibbs sampling, and the EM algorithm, for parameter estimation. Our findings, based on real-world data examples of GC patients, demonstrate that the Gibbs sampler method yields significantly improved prediction accuracy compared to the EM algorithm. The application of Gibbs sampling in our study has shown superior performance in accurately predicting outcomes, indicating its effectiveness in modeling and analyzing the given dataset. These results underscore the value of incorporating Gibbs sampling as a powerful tool for enhancing prediction accuracy in real-world scenarios involving GC patients. On the other hand, the simulation study revealed that NB-BLCA based on the EM algorithm was superior to the ordinary NB classifier in all the predefined scenarios. However, we should admit that our model is more sophisticated than the standard NB classifier in structure. Therefore, the usual trade-off between complexity and accuracy matters here. However, attention to the properties of each algorithm facilitates the fitting procedure and leads to more accurate results.

In the context of adjusting the naive Bayesian classifier when the conditional assumptions are violated, latent variable models emerge as one of the optimal solutions [4, 41]. This assumption often fails to capture complex relationships and dependencies among features, leading to suboptimal performance. To overcome these limitations, latent variable models offer a powerful framework. By introducing latent variables, these models can capture the hidden dependencies and relationships among features, even in cases where the conditional independence assumption is violated [3]. The inclusion of latent variables allows for more flexible and expressive modeling, enabling the representation of intricate interactions among features [3].

One key advantage of latent variable models is their ability to handle missing data and incomplete feature sets [42]. By incorporating latent variables, these models can effectively impute missing values, mitigating the impact of incomplete information on classification accuracy. This is particularly valuable in real-world scenarios where data may be incomplete or contain missing values [43]. Furthermore, latent variable models provide a means to account for unobserved or latent factors that may influence the observed features [44]. By capturing these latent factors, the models can better explain the underlying data distribution and improve classification performance.

Another benefit of latent variable models is their ability to offer principled probabilistic inference [45]. This allows for robust uncertainty quantification and provides richer insights into the model's predictions. By understanding the uncertainty associated with the predictions, decision-makers can make more informed choices based on the level of confidence or uncertainty in the classification results.

In summary, when the conditional assumptions of the naive Bayesian classifier are violated, latent variable models serve as an optimal solution. By incorporating latent variables, these models capture hidden dependencies, handle missing data, account for unobserved factors, and offer principled probabilistic inference. Their ability to address the limitations of the naive Bayesian classifier makes latent variable models a valuable tool for improving classification performance in scenarios where conditional assumptions are not met.

The Gibbs sampler is one of the most efficient and well-known MCMC algorithms. This algorithm is a special case of Metropolis-Hasting sampling wherein the randomly generated values are always accepted. It works based on the Markov property and generates random samples from the univariate conditional posterior distributions instead of an expensive joint distribution [35, 46]. Therefore, the Gibbs sampler leads to the answers more quickly and needs less computational complexity. However, the samples achieved from this approach still are highly correlated. In this situation, thinning the samples has been suggested to make samples independent. It means picking separated points from the generated chain systematically [47]. Separating the samples from the Markov chain dilutes the dependency and makes them independent. Another drawback of MCMC methods is the impact of misspecification of the initial values on the convergence of the chain. Fortunately, in most cases, the chain corrects itself at each scan, and we ensure that the later samples reflect the actual posterior distribution [48]. Therefore, the only task we need is to burn in the initial values of the chain. Typically references suggest a basic rule of the first 1000 to 5000 sample burn-in [49]. The other proposes a more conservative approach to selecting the starting value close to the distribution mode achieved from a likelihood-based model [50]. We can use all these considerations to ensure chain convergence by correctly tuning the parameters.

As we confronted here, the EM algorithm is widespread in the case of the mixture distribution [51, 52]. However, such a method is not without drawbacks. For instance, there is no guarantee to achieve global optima. In addition, the real value near the boundary makes the estimations unstable. Using parametric bootstrap sampling and refitting the model could benefit these situations [30]. Hence, we restarted all the processes in the EM algorithm ten times in the simulation study and real-world data example. This approach is not straightforward when we sample from low-probability groups. To overcome this problem, using likelihood sampling and logic sampling methods have been proposed [53]. Fortunately, due to appropriate prior distribution, Gibbs's sampler is not a case of this issue. In this study, Beta and Dirichlet priors are proper and conjugate for parameters of interest [54].

The NB-BLCA model needs to determine the number of latent class variables and the number of levels for each of them. Data gathering in many medical and health applications starts after determining risk factors, influential predictors, and related domains [5]. Therefore, the specialist could supervise us in detecting the required latent variables. However, it is not a general rule, especially in data mining applications. More development seems necessary in this situation. On the other hand, the number of levels for each latent variable depends on the data. Like principal component analysis (PCA) and Explanatory Factor Analysis (EFA), the best choice of levels could be made using the scree plot [55]. In this manner, AIC and BIC criteria for both Gibbs sampling and EM algorithm and DIC for Gibbs sampling could lead us to select the best choice.

## Conclusion

The addition of a latent component to the NB classifier model offers numerous advantages when compared to other modification attempts. Firstly, it aligns well with the nature of the data, particularly within medical and health contexts. Furthermore, incorporating the latent component allows us to bypass the extensive search algorithm and structure learning required in the local learning and structure extension approach. By utilizing latent class variables, all attributes are incorporated into the model building process, unlike attribute selection approaches that may ignore certain variables and result in the loss of information. As a result, the NB-BLCA model emerges as a suitable alternative to ordinary NB classifiers, particularly when the assumption of independence is violated, especially in the domains of health and medicine.

### Supplementary Information

**Additional file 1:** **S-Table 1.** List of questionnaire binary attributes with the categories used in the Real-world data example.

## Data Availability

The datasets used or analyzed during the current study are available from the corresponding author upon reasonable request.

## Abbreviations

NB: Naïve Bayes
BLCA: Bayesian Latent Class Analysis
EM: Expectation Maximization
AIC: Akaike Information Criterion
BIC: Bayesian Information Criterion
DIC: Deviance Information Criterion
NCII: National Cancer Institute of Iran
GC: Gastric Cancer
NUD: Non-ulcer dyspepsia
ML: Machine Learning
ANB: Augmented Naive Bayes
TAN: Augmented Naive Bayes
eTAN: extended Tree Augmented Naive Bayes
AODE: Averaged One-Dependence Estimators
