# Supplementary material for: A Bayesian latent class extension of naive Bayesian classifier and its application to the classification of gastric cancer patients
Source: BMC Med Res Methodol. 2023 Aug 21;23:190. doi: 10.1186/s12874-023-02013-4 (PMC10440900; doi:10.1186/s12874-023-02013-4)
Supplement: Supplementary file 1 — Additional file 1: S-Table 1. List of questionnaire binary attributes with the categories used in the Real-world data example. [file 12874_2023_2013_MOESM1_ESM.doc]

S-Table . List of questionnaire binary attributes with the categories used in the Real-world data example.

| **Predictors** | | **level 0** | **Level 1** |
| --- | --- | --- | --- |
| **1. Demographic** | | | |
| 1 | Age | **≤60** | **>60** |
| 2 | Ethnicity | **Fars** | **Non-Fars (Non-Fars/ mixed)** |
| 3 | Gender | **Female** | **Male** |
| **2. Diet** | | | |
| 4 | Canned food | **Never** | **Ever** (1/day, 5-6/week, 3-4/week, 1-2/week, 2-3/month, bimonthly, 1/month, 2-3/year) |
| 5 | Carbonated soft drinks | **Never to low** (never, 1/month, 2-3/month) | **Medium to high** (>5/day, 3-5/day, 2/day, 1/day, 5-6/week, 3-4/week, 1-2/week) |
| 6 | Cheese | **Never to low** (never, 1/month, 2-3/month, 1-2/week) | **Medium to high** (>5/day, 3-5/day, 2/day, 1/day, 5-6/week, 3-4/week) |
| 7 | Chicken | **Never to low** (never, 1/month, 2-3/month) | **Medium to high** (>5/day, 3-5/day, 2/day, 1/day, 5-6/week, 3-4/week, 1-2/week) |
| 8 | Coffee | **Never to low** (never, 1/month, 2-3/month, 1-2/week, 3-4/week, 5-6/week) | **Medium to high** (>5/day, 3-5/day, 2/day, 1/day) |
| 9 | Cooking method | **Boiling** | **Other than boiling (**Frying, grilled, steamed, microwave, roasting**)** |
| 10 | Cooking oil | **Unsaturated** | **Saturated or both** |
| 11 | Cooking salt | **Never to low** | **Medium to high** |
| 12 | Dinner time | **Early** (early evening) | **Late** (late evening) |
| 13 | Drinking water (childhood) | **City plumbing** | **Other** (well, river, pipe, water tank) |
| 14 | Egg | **Never to low** (never, 1/month, 2-3/month) | **Medium to high** (>5/day, 3-5/day, 2/day, 1/day, 5-6/week, 3-4/week, 1-2/week) |
| 15 | Fish | **Never to low** (never, 1/year, 2/year, 3/year, 4/year, 1/month, bimonthly, 2-3/month) | **Medium to high** (2/day, 1/day, 5-6/week, 3-4/week, 1-2/week) |
| 16 | Fruit | **Never to low** (never, 1/month, 2-3/month, 1-2/week) | **Medium to high** (>5/day, 3-5/day, 2/day, 1/day, 5-6/week, 3-4/week) |
| 17 | Milk | **Never to low** (never, 1/month, 2-3/month, 1-2/week, 3-4/week) | **Medium to high** (3-5/day, 2/day, 1/day, 5-6/week) |
| 18 | Minerals | **Never** | **Ever** (3-5/day, 2/day, 1/day, 5-6/week, 3-4/week, 1-2/week, 2-3/month, 1/month) |
| 19 | Pickled vegetables | **Never to low** (never, 1/month, 2-3/month) | **Medium to high** (3-5/day, 2/day, 1/day, 5-6/week, 3-4/week, 1-2/week) |
| 20 | Processed meats | **Never to low** (never, 1/month, 2-3/month) | **Medium to high** (3-5/day, 2/day, 1/day, 5-6/week, 3-4/week, 1-2/week) |
| 21 | Potato chips | **Never** | **Ever** (2/day, 1/day, 3-4/week, 1-2/week, 2-3/month, 1/month) |
| 22 | Red meat | **Never to low** (never, 1/month, 2-3/month, 1-2/week) | **Medium to high** (>5/day, 3-5/day, 2/day, 1/day, 5-6/week, 3-4/week) |
| 23 | Salted food | **Never** | **Ever** |
| 24 | Smoked fish | **Never** | **Ever** (2/day, 1/day, 3-4/week, 1-2/week, 2-3/month, 1/month, 2/year, 1/year) |
| 25 | Smoked rice | **Never** | **Ever** (3-5/day, 2/day, 1/day, 3-4/week, 1-2/week, 2-3/month, 1/month) |
| 26 | Table salt | **Never** | **Low to high** |
| 27 | Tea | **Never to low** (never, 1/month, 2-3/month, 1-2/week, 3-4/week, 5-6/week) | **Medium to high** (>5/day, 3-5/day, 2/day, 1/day) |
| 28 | Tea temperature | Cold to warm | Hot |
| 29 | Tuna fish | **Never** | **Ever** (3-5/day, 2/day, 1/day, 3-4/week, 1-2/week, 2-3/month, 1/month, 2/year, 1/year) |
| 30 | Vegetable | **Never to low** (never, 1/month, 2-3/month, 1-2/week) | **Medium to high** (3-5/day, 2/day, 1/day, 5-6/week, 3-4/week) |
| 31 | Vitamin | **Never** | **Ever** (3-5/day, 2/day, 1/day, 3-4/week, 1-2/week, 2-3/month, 1/month) |
| 32 | Yoghurt | **Never to low** (never, 1/month, 2-3/month, 1-2/week) | **Medium to high** (3-5/day, 2/day, 1/day, 5-6/week, 3-4/week) |
| **3. Medical status (self-reported)** | | | |
| 33 | Colitis | **No** | **Yes** |
| 34 | Diabetes | **No** | **Yes** |
| 35 | Esophageal reflux | No | Yes |
| 36 | Esophagitis | No | Yes |
| 37 | Family history of GC | **No** | **Yes** |
| 38 | Family history of GI cancers | **No** | **Yes** |
| 39 | Family history of stomach operation | **No** | **Yes** |
| 40 | Fatty liver | **No** | **Yes** |
| 41 | Gastritis | **No** | **Yes** |
| 42 | Personal history of GI cancers | No | Yes |
| 43 | Stomach operation | **No** | **Yes** |
| **4. Narcotics** | |  |  |
| 44 | Alcohol | **Never** | **Ever** (casual, current, former) |
| 45 | Opium | **Never** | **Ever** (casual, current, former, exposed) |
| 46 | Smoking | **Never** | **Ever** (current, former, exposed) |
| 47 | Waterpipe | **Never** | **Ever** (casual, current, former) |
| 48 | Passive smoking (childhood) | **Never** | **Ever** (exposed with cigarette/water pipe) |
| **5. Socioeconomic status (SES)** | | | |
| 49 | Birth place | **Urban** | **Rural** |
| 50 | Chemical exposure | **Never** | **Ever** (respiratory, dermal, unknown) |
| 51 | Crowdedness | **≤ 2** | **> 2** |
| 52 | Crowdedness (childhood) | **≤ 2** | **> 2** |
| 53 | Drinking water | **City plumbing** | **Other than city plumbing** (well, river, pipe, water tank) |
| 54 | Education | > 8yrs | < 8yrs |
| 55 | Job-related physical activities | **High (**freelancer, laborer, office employee, student) | **Low (**house keeper, retired, unemployed) |
| 56 | Marital status | **Other** (divorced, single, widow) | **Married** |
| 57 | Parents illiteracy (both) | **No** | **Yes** |
| 58 | Physical exercise per week | **Never** | **Ever** (1-2/week, 3-5/week, everyday) |
| 59 | Refrigerator use | **Yes** | **No** |
| 60 | Refrigerator use (childhood) | **Yes** | **No** |
| 61 | Residence place | **Urban** | **Rural** |
| 62 | Residence place (childhood) | **Urban** | **Rural** |
| 63 | Residence type | **Owned** | **Rented** |
| 64 | Residence type (childhood) | **Owned** | **Rented** |
